# Comparison and optimization of conventional and ultrasound‐assisted solvent extraction for synthetization of lemongrass (*Cymbopogon*)‐infused cooking oil

**DOI:** 10.1002/fsn3.2234

**Published:** 2021-03-22

**Authors:** Soh Fong Lim, Adirah Hamdan, Sing Ngie David Chua, Bee Huah Lim

**Affiliations:** ^1^ Faculty of Engineering Universiti Malaysia Sarawak Kota Samarahan Malaysia; ^2^ Fuel Cell Institute Universiti Kebangsaan Malaysia Bangi Malaysia

**Keywords:** conventional solvent extraction, infused oil, lemongrass, lemongrass‐infused cooking oil, optimization, ultrasound‐assisted solvent extraction

## Abstract

The lemongrass plant, which is widely cultivated in Asia, Australia, and Africa, has been reported to have many significant health benefits such as antimicrobial, insecticide, anticancer, fight fever, and disinfection. Therefore, it is an added benefit to have lemongrass compounds in cooking oil. This study was aimed to compare the conventional (CSE), and ultrasound‐assisted solvent extraction (UASE) for citral compounds from lemongrass (*Cymbopogon*) leaves and to optimize the best extraction method using the response surface methodology (RSM) and ANOVA. RSM design of experiments using three types of cooking oils; palm oil, sunflower oil, and corn oil. The effect of three independent variables, which are temperature (48.2–81.8°C), extraction time (4.8–55.2 min), and solvent to leaves ratio (5.3–18.7), was investigated. The characterization of lemongrass‐infused cooking oil was evaluated by Fourier transform infrared spectroscopy (FT‐IR), Gas Chromatography‐Mass Spectrometry (GC‐MS) and Scanning Electron Microscopy (SEM) analysis for confirmation of the citral compound extraction. This extraction process is optimized using Response Surface Methodology (RSM) for producing the lemongrass‐infused cooking oil. After optimization, the UASE process gives 1.009 × 10^6^ maximum citral area for palm oil and 1.767 × 10^6^ maximum citral area for sunflower oil. CSE process only can give 2.025 × 10^5^ and 2.179 × 10^5^ citral area in the GC‐MS spectrum for palm oil and sunflower oil respectively. For both the UASE and the CSE, the optimum operating conditions are 81.8°C of extraction temperature and 55.2 min of extraction time except for lemongrass‐infused palm oil in the CSE process with 45 min extraction time. The optimum solvent to leaves ratio varies from 5.3:1 to 12.9:1. This study found that corn oil cannot be used as a solvent to extract lemongrass‐infused cooking oil due to the insignificant changes and no citral peak. The lemongrass (Cymbopogon)‐infused palm oil and sunflower oil extracted using the UASE have a higher maximum citral area than the CSE process.

## Graphical abstract

This study was aimed to compare the conventional (CSE) and ultrasound‐assisted solvent extraction (UASE) for citral compounds from lemongrass (Cymbopogon) leaves and to optimize the best extraction method using the response surface methodology (RSM) and ANOVA. A quadratic model was proposed to correlate the independent variables for maximum citral extraction at the optimum process condition by using central composite design (CCD) method. The characterization of lemongrass‐infused cooking oil was evaluated by Fourier transform infrared spectroscopy (FT‐IR), Gas Chromatography‐Mass Spectrometry (GC‐MS), and Scanning Electron Microscopy (SEM) analysis for confirmation of the citral compound extraction.

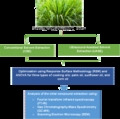

## 1 INTRODUCTION

Lemongrass is a native and widely cultivated in tropical and sub‐tropical climates of Asia, Australia, and Africa and is usually used as an ingredient in cooking (Singh et al., 2014). An extraction process can produce lemongrass oil from its leaves. An ancient India has used it to fight fever and infection (Al‐shaer, 2006). Lemongrass oil is characterized by having a significant component of citral, which contains a mixture of tran‐citral and cis‐citral (Weisheimer, 2008). It was reported to have many advantages such as antimicrobial activities, insecticide activity, and anticancer (Avoseh et al., 2015). Hence, various extraction process of lemongrass oil may give many advantages to pharmaceutical industries.

Many methods can extract volatile oil from different parts of a plant such as leaves, fruit, flower, and stem (Falcão et al., 2012). Extraction methods such as solid‐liquid extraction, also known as Soxhlet, distillation, enfleurage, and maceration, were traditionally used (Kostova et al., 2010). However, there new extraction methods. such as Accelerated Solvent Extraction (ASE), Ultrasound‐Assisted Solvent Extraction (UASE), Microwave‐Assisted Extraction (MAE) and Supercritical Fluid Extraction (SCFE), had been introduced (Carlson et al., 2001; Falcão et al., 2012; Kaur & Dutt, 2013; Lim et al., 2017; Parniakov et al., 2015; Petigny et al., 2013; Sodeifian, Ardestani, et al., 2016; Sodeifian, Sajadian, et al., 2016). The new extraction technologies can shorten the extraction time, reduce solvent consumption, increase pollution prevention, and higher consent for thermolabile constituents (Shams et al., 2015). The SCFE method is an extraction technique using fluids in elevated conditions above their critical point of temperature (Sodeifian, Ardestani, et al., 2016; Sodeifian, Sajadian, et al., 2016). Carlson et al. (2001) extracted the lemongrass (Cymbopogon citratus) essential oil using dense carbon dioxide at 23–50°C and 85–120 bar. The requirement for high pressures in the SCFE increases the cost compared to conventional liquid extraction. The usage of carbon dioxide as a solvent in the SCFE is non‐polar and has limited dissolving power, which cannot always be used as a solvent on its own, particularly for polar solutes.

The UASE method is cheaper than other advanced extraction techniques, and its operation is much easier (Wang & Weller, 2006). This method induced cavitation bubbles and accelerates the release of organic compounds contained within the plant body. Thus, the yield of oil obtained can be increased (Parniakov et al., 2015). The design of green and sustainable extraction methods of natural products is currently a hot research topic in the multidisciplinary area of applied chemistry, biology, and technology (Chemat et al., 2012). Hence, a green solvent can be used as alternative solvents or innovatory plant resources and eliminate petroleum‐based solvents to ensure high quality extracted products (Li et al., 2014). In conjunction, many researchers used extracted products as value‐added or additives in food industries. Thus, various types of cooking oil were used so that the oil base used in cooking is full of nutrients. This study was aimed to compare the conventional (CSE) and ultrasound‐assisted solvent extraction (UASE) for citral compounds from lemongrass (*Cymbopogon*) leaves and to optimize the best extraction method using the response surface methodology (RSM) and ANOVA using three types of cooking oils; palm oil, sunflower oil, and corn oil. This paper focuses on examining the optimum conditions for synthetization of lemongrass (*Cymbopogon*)‐infused cooking oil and the information derived is particularly important for scaling up the operation.

## 2 MATERIALS AND METHODS

### 2.1 Materials

All chemicals which were utilized in this study were of analytical grade. Distilled water was consumed in the preparation of solutions. The lemongrass (Cymbopogon) leaves were collected from a residential area of Kota Samarahan, Sarawak, Malaysia. Cooking oils such as sunflower oil, corn oil, and olive oil were purchased from the consumer market.

To extract the lemongrass oil, an ultrasonic cleaning bath and heating bathtub were used. Moreover, GC‐MS and FT‐IR were used for chemicals analysis purposes. Other than that, to analyse the oil's physical properties, Portable Density Meter DMA 35 and Atago 3850 Refractometer were used.

### 2.2 Experimental design

Response surface methodology (RSM) based on the circumscribed central composite design (CCD) was employed to study the effect of the three independent variables; time, temperature, and solvent to lemongrass leaves ratio ( Lim et al., 2017). Each variable was scrutinized at five following levels: −1, −*α*, 0, +*α*, and +1, as tabulated in Table 1. These three independent variables were studied in a multivariate study with 20 experimental runs. As described in Equation 1, the empirical quadratic model explains the behavior of the extraction process.

(1) $$Y=\beta_{1}A+\beta_{2}B+\beta_{3}C+\beta_{12}AB+\beta_{13}AC+\beta_{23}BC+\beta_{11}A^{2}+\beta_{22}B^{2}+\beta_{33}C^{2}+\alpha$$

where, *Y* = predicted response; *A*, *B* and *C* = uncoded factors; *β* = coefficients; *α* = offset term.

**TABLE 1:** The coded and uncoded level of three selected independent variables used in the study

Selected independent variables	Symbol	Unit	Coded and uncoded levels				
			−1	−*α*	0	+*α*	1
Temperature	T	^o^C	48.2	55	65	80	81.8
Time	t	min	4.8	15	30	45	55.2
Solvent to lemongrass leaves ratio	S/L	ml/g	5.3	8	12	16	18.7

The response surface fittings were generated using MATLAB version R2019b to enable a surface fitting analysis of the response over an entire range of varying factors with 20 number of experimental runs. Data were analysed using variance (ANOVA) analysis, and the effective treatment means were separated by the least significant difference (LSD) at a 95% confidence level. The relationships between the citral area in the GC‐MS chromatogram and all parameter were determined using regression analysis. All statistical analysis was performed using Minitab Statistical Software.

### 2.3 Extraction procedures

The extraction procedures were carried out using both CSE and UASE methods to optimize the extraction operating conditions. The lemongrass leaves have undergone two processes: ultrasound‐assisted solvent extraction (UASE) and conventional solvent extraction (CSE). The extracts were aliquoted and analysed using instrumental analyses.

#### 2.3.1 Conventional solvent extraction

The experiments were conducted for the conventional solvent extraction using the lemongrass leaves, which were cut and weighed according to the fixed parameter in a 250 ml beaker. One hundred milliliter of solvent was measured and mixed with the weighed lemongrass leaves. The sample was then blended and subjected to treatment in an indirect heating bath with the time and temperature established in the experimental design. The extract and leaves mixture was filtered on a sieve to separate the leaves and extracted product. The extracted product was then stored in the dark and under room temperature (24°C) until used.

#### 2.3.2 Ultrasound‐assisted solvent extraction

The extraction of lemongrass oil was performed in an ultrasound bath (Ultronique, Q 5.9/40A, Eco‐Sonics), with the power of 176 W and ultrasound frequency of 100 kHz. In each experimental run, fresh lemongrass leaves were cut and weighed according to the 250 ml beaker's fixed parameter. Next, 100 ml of solvent was measured and mixed with the weighed lemongrass leaves. The sample was then blended and subjected to treatment in an indirect bath ultrasound with the time and temperature established in the experimental design. The extract and leaves mixture was filtered on a sieve to separate the leaves and extracted product. The extracted product was then stored in the dark and under room temperature (24°C) until further usage.

### 2.4 Instrumental analysis of the lemongrass‐infused cooking oil

#### 2.4.1 Scanning electron microscopy (SEM)

The SEM analysis was conducted using a Hitachi TM3030 Tabletop Microscope to determine the lemongrass leaf samples' surface morphology for both after CSE and UASE processes respectively. The samples were coated with Au film and mounted onto a stub which was placed inside the SEM equipment. Micrographs of the samples with different magnifications were recorded from the SEM equipment for further analysis.

#### 2.4.2 Fourier transform infrared spectroscopy (FT‐IR)

The functional groups present in the lemongrass‐infused cooking oil were defined by comparing the FT‐IR spectrum's wavelengths with an infrared (IR) correlation chart from literature. The FT‐IR spectrometer (Iraffinity‐1, Shimadzu) was used to obtain an infrared spectrum of the samples absorption. The samples were placed directly on the Attenuated Total Reflection (ATR) top plate at room temperature. The measurements were performed in the IR region intensity over a range of wavelengths from 4,000 to 600 cm^−1^. All of the measurement scannings were performed at a resolution of 4.00 cm^−1^ with a scanning speed of 0.20 cm/s.

#### 2.4.3 Gas chromatography‐mass spectrometry (GC‐MS)

A gas chromatograph (Shimadzu GP‐2010) coupled to mass spectrometry (GC‐MS) was used to analyse the chemicals component in the lemongrass‐infused cooking oil. The GC‐MS is equipped with a 30 m × 0.25 mm non‐polar DPX‐5 capillary column with 0.25 μm film thickness. The column temperature was set at 80°C for 2 min, increased at 10°C per minute, and held at 260°C for 18 min. The injector and detector temperatures were set at 200 and 280°C respectively. Helium gas was used as the carrier gas at a flow rate of 1.0 ml/min. 1.0 μl of diluted lemongrass oil (1/10 in HPLC‐grade Hexane) was injected automatically and splitless. Identifying components was based on the 2008 version of the NIST/EPA/NIH Mass Spectral Library (NIST 08).

## 3 RESULTS AND DISCUSSION

### 3.1 RSM model development

Three types of solvents (palm oil, sunflower oil, and corn oil) were used to examine the effects of extraction solvent on citral compound using both the CSE and UASE. The multivariate study was done for each type of oil and technique of the extraction used. The optimizing process was done using Minitab Statistical Software to predict the extraction process trend and its optimum condition. The citral area data from the GC‐MS were recorded to represent the concentration of citral compounds in each sample. Table 2 summarizes the RSM design for the three‐independent variables and five‐levels and the scrutinized responses on citral compounds under different experimental conditions. Although all 20 multivariate studies for both UASE and CSE process were carried out, it is found out that corn oil did not give citral area in the GC‐MS spectrum. The GC‐MS spectrum of all of the samples for both processes did not show the citral peak after the UASE and CSE process. Hence, it is concluded that corn oil cannot be used as a solvent to extract lemongrass‐infused cooking oil.

**TABLE 2:** A RSM design for three‐variables and five‐levels and scrutinized responses under different experimental conditions on citral compounds

Experiment number	Temperature T (^o^C)	Time t (min)	Solvent to lemongrass leaves ratio S/L (ml/g)	Citral area (mAu.min)					
				Palm oil		Sunflower oil		Corn oil	
				CSE	UASE	CSE	UASE	CSE	UASE
1	65.0	30.0	12.0	148,866	201,244	65,440	261,855	0	0
2	65.0	30.0	18.7	127,150	105,350	65,948	127,150	0	0
3	65.0	55.2	12.0	141,537	399,504	73,490	371,132	0	0
4	55.0	15.0	8.0	52,240	201,629	59,821	507,214	0	0
5	65.0	30.0	12.0	112,397	219,495	76,725	333,729	0	0
6	65.0	30.0	12.0	128,302	221,901	69,421	343,223	0	0
7	65.0	4.8	12.0	70,607	98,745	35,293	527,079	0	0
8	65.0	30.0	12.0	127,500	210,465	57,478	231,739	0	0
9	75.0	45.0	16.0	116,867	475,257	103,336	986,059	0	0
10	55.0	45.0	8.0	64,240	120,168	68,940	371,736	0	0
11	55.0	15.0	16.0	39,932	75,941	25,356	172,300	0	0
12	65.0	30.0	12.0	147,002	204,074	68,718	311,666	0	0
13	75.0	45.0	8.0	178,896	600,080	159,172	1,149,057	0	0
14	65.0	30.0	12.0	100,701	263,570	51,532	223,291	0	0
15	81.8	30.0	12.0	177,838	562,148	154,031	562,148	0	0
16	55.0	45.0	16.0	46,840	76,908	34,317	126,837	0	0
17	48.2	30.0	12.0	51,785	96,155	49,946	256,114	0	0
18	75.0	15.0	8.0	149,385	331,739	138,275	221,907	0	0
19	65.0	30.0	5.3	102,738	366,468	51,172	231,739	0	0
20	75.0	15.0	16.0	103,039	285,340	98,016	220,520	0	0

**FIGURE 1:**
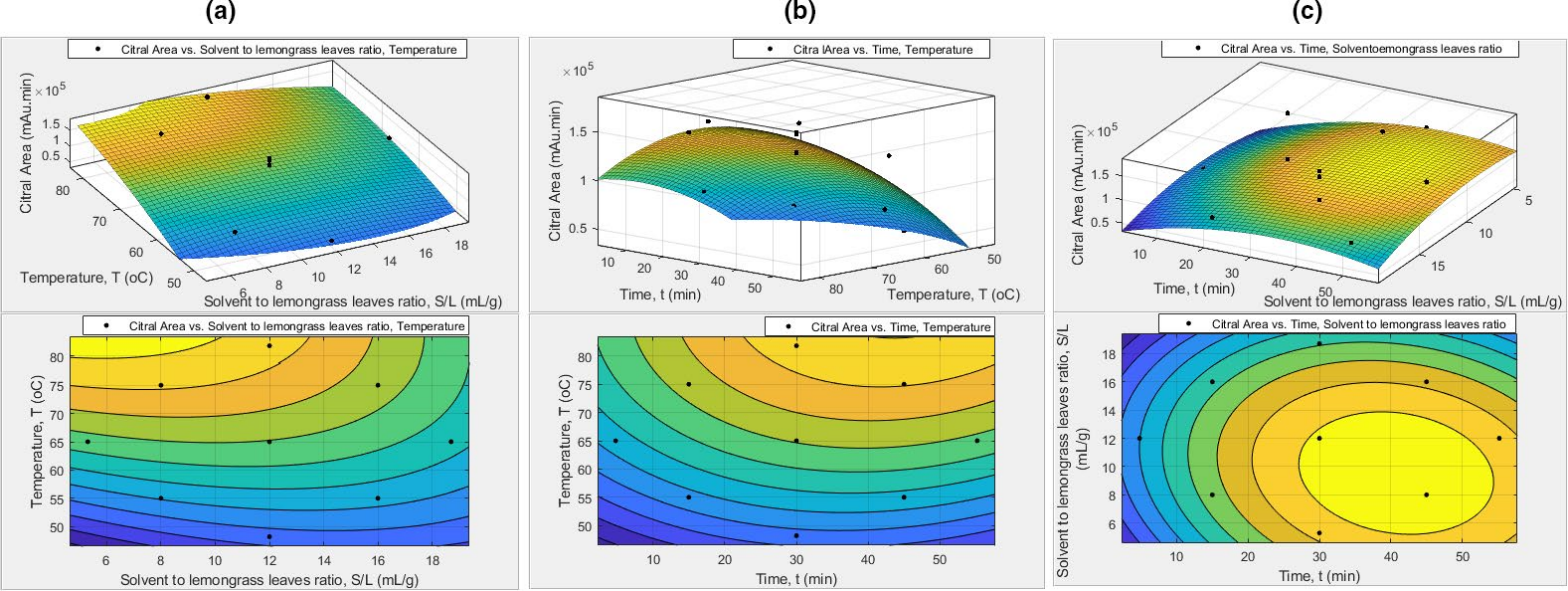
Effects of palm oil as solvent using CSE. The response surface and contour plots of: (a) citral area versus temperature and time; (b) citral area versus temperature and solvent to lemongrass leaves ratio; and (c) citral area versus time and solvent to lemongrass leaves ratio

**FIGURE 2:**
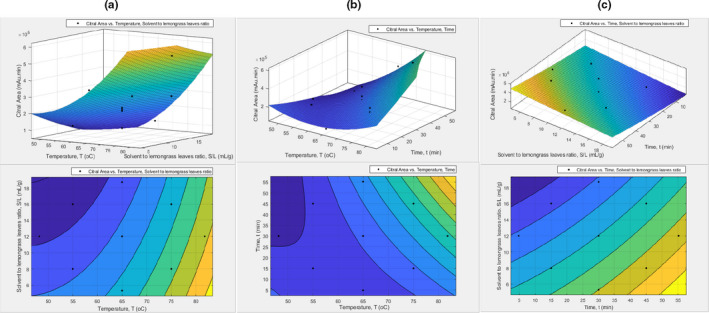
Effects of palm oil as solvent using UASE. The response surface and contour plots of: (a) citral area versus temperature and time; (b) citral area versus temperature and solvent to lemongrass leaves ratio; and (c) citral area versus time and solvent to lemongrass leaves ratio

**FIGURE 3:**
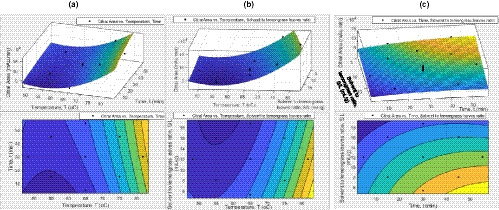
Effects of sunflower oil as solvent using CSE. The response surface and contour plots of: (a) citral area versus temperature and time; (b) citral area versus temperature and solvent to lemongrass leaves ratio; and (c) citral area versus time and solvent to lemongrass leaves ratio

**FIGURE 4:**
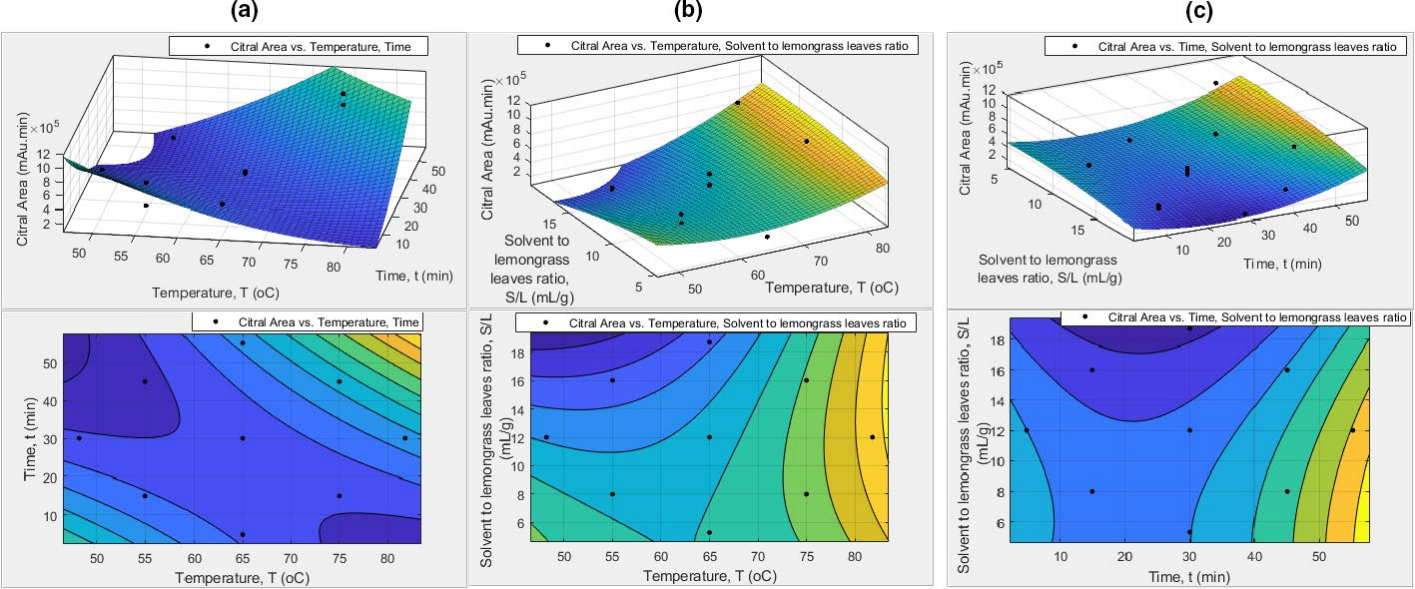
Effects of sunflower oil as solvent using UASE. The response surface and contour plots of: (a) citral area versus temperature and time; (b) citral area versus temperature and solvent to lemongrass leaves ratio; and (c) citral area versus time and solvent to lemongrass leaves ratio

By using Matlab and Minitab Software, both CSE and UASE processes' operating conditions are optimized and analysed for the effects of palm oil and sunflower oil as the solvent. The relationship for each parameter is shown in Figures 1, 2, 3, 4. The temperature versus solvent to leaves ratio graph (Figure 1a) for palm oil as solvent using the CSE shows that a low solvent to leaves ratio gives a higher mean of citral area. This is due to the concentration of the citral is decreasing as the solvent to leaves proportion increasing. The temperature versus time graph (Figure 1b) shows that the mean of citral area is growing as the temperature and time increasing for palm oil solvent.

At the beginning of extraction, 45 min of extraction duration time gives less citral area than 30 min. However, as the temperature increases, at the 45 min of an extraction duration time, a high amount of mean citral area is obtained. Moreover, the time versus solvent to leaves ratio graph (Figure 1c) shows that the solvent to leaves ratio of 16 gives a lower mean of citral area than 8 and 12 ratios. Besides that, it is shown that the mean of citral area is increasing as the duration of the extraction process increased. When the extraction time is higher, the time for the citral infused into the solvent is expanding. Hence, the amount of extracted lemongrass oil is increased. Table S1 in the supplementary section shows the optimum condition of the CSE process for palm oil. It is estimated that at 81.8°C, 45 min, and 5.4:1 of solvent to leaves ratio as the process's optimum operating conditions. It gives a maximum citral area which is 2.025 × 10^5^ citral area in the GC‐MS spectrum.

Figure 2 shows the interaction effects of temperature, time, and solvent to leaves ratio on citral area using palm oil as a solvent in the UASE. The mean of citral area is increasing as the temperature increasing. The solvent to leaves ratio versus temperature graph (Figure 2a) reveals that the lower the solvent to leaves ratio, the higher the mean of citral area. This relationship might be due to the concentration of the citral is declining as the liquid to solid amount heaped. The temperature versus time graph (Figure 2b) shows that at the low temperature of extraction, 45 min of extraction duration gives less mean of citral area compared to 15 min. However, as the temperature increases, the 45 min of extraction duration getting a higher mean of citral area.

Moreover, Figure 2c shows that the S/L ratio of 8 and below gives a higher mean of citral area. It is also demonstrated that the mean of citral area is rising as the duration time of extraction getting big, which insinuates that the extraction duration time is proportional to the time for the citral compounds to infuse into the solvent. The optimum condition of the UASE process for palm oil is unfolded in Table S1. The estimated optimum operating condition is at the temperature of 81.8°C for 55.2 min with a solvent to leaves ratio of 5.3:1, which produced a maximum citral area of 1.009 × 10^6^ mAu.min citral area in the GC‐MS spectrum.

Figures 3 and 4 show the response surface and contour plots for the effects of sunflower oil as solvent using CSE and UASE respectively. The temperature versus time graph in Figures 3a and 4a shows that the citral area's mean increases as the temperature rises. However, the 15 min duration of UASE extraction decreases over the increasing temperature (Figure 4a). The temperature versus solvent to leaves ratio graphs (Figures 3b and 4b) show that the lower the solvent to leaves ratio gives a higher mean of citral area. This is due to the concentration of the citral is decreasing as the solvent to leaves proportion increasing.

Moreover, the time versus solvent to leaves graph Figure 4c displays that the solvent to leaves ratio of 8 gives a higher mean of citral area. Besides that, it is delineated that the mean of the citral area increases as the duration of extraction increases. When the extraction duration is higher, the time for the citral infused into the cooking oil is rising. Hence, the amount of extracted lemongrass oil is increased. It is estimated that at 81.8°C, 55.2 min and 5.3:1 of solvent to leaves ratio, the optimum condition of the CSE process using the sunflower oil give a maximum citral area which is 2.179 × 10^5^ GC‐MS spectrum citral area. The optimum condition of the UASE process for sunflower oil as laid out in Table S1 is at 81.8°C, 55.2 min and 12.9:1 of solvent to leaves ratio which produced a maximum citral area of 1.767 × 10^6^ citral area in the GC‐MS spectrum.

The significant model terms for lemongrass‐infused cooking oil are the main effect of temperature (A), the main effect of extraction time (B), the main effect of solvent to leaves ratio (C), the two‐level interactions of temperature and temperature (AA), the two‐level interactions of extraction time and extraction time (BB), the two‐level interactions of solvent to leaves ratio and solvent to leaves ratio (CC), the two‐level interactions of temperature and extraction time (AB), the two‐level interactions of temperature and solvent to leaves ratio (AC), and the two‐level interactions of extraction time and solvent to leaves ratio (BC). The mathematical relationship between the variables and the response is expressed by the second‐order polynomial equation, tabulated in Table 3. In ensuring a good model for the response, two tests were performed to fit the regression model; (a) significance on individual coefficients and (b) lack‐of‐fit. Fully coded experiments and responses obtained for each run of the CCD were tabulated in Table 4. The significance and suitability of the optimized conditions were then studied using analysis of variance (ANOVA). Statistical significance of each effect, including interaction terms, linear and quadratic impact, was validated by comparing the mean squared against estimated experimental error. Depending upon the degree of freedom (*df*) involved, *F*‐ratio can be calculated (the mean squared error to the pure error). With a confidence level of 95%, *F*‐ratio significance is evaluated using the *p*‐value column. In this column, when the value is lower than 0.05, the effect is significant. Tables 3 and 4 show that all three parameters significantly affect the extraction process. Other than that, *R* ^2^ represents the proportion of the total variability explained by the regression model. It is a measure for the amount of response variation defined by the variables and will always increase when a new term is added to the model. The $R_{\text{adj}}^{2}$ is an adjusted form of *R* ^2^ for several terms in the model. The *R* ^2^ and $R_{\text{adj}}^{2}$ values are relatively high and are at an acceptable range.

**TABLE 3:** Mathematical relationship between the variables and the response using second‐order polynomial model

Solvent	Process	Mathematical equation	Equation No.
Palm oil	CSE	$\begin{array}{cc} \text{Citral}\;\text{Area}= & ‐768,035+16,915\times A+3,059\times B+27,543\times C‐80.7\times AA\\ & ‐49.6\times BB‐502\times CC+20.4\times AB‐246\times AC‐43\times BC \end{array}$	2
	UASE	$\begin{array}{cc} \text{Citral}\;\text{Area}= & 3,831,125‐102,812\times A‐34,328\times B‐52,763\times C+800.6\times AA\\ & +143.7\times BB+1,728\times CC+449.0\times AB‐7\times AC+8\times BC \end{array}$	3
Sunflower oil	CSE	$\begin{array}{cc} \text{Citral}\;\text{Area}= & 464,635‐16,488\times A+693\times B+2,872\times C+160.4\times AA\\ & ‐3.5\times BB+43\times CC+6.8\times AB‐84\times AC‐33\times BC \end{array}$	4
	UASE	$\begin{array}{cc} \text{Citral}\;\text{Area}= & 6,582,315‐139,372\times A‐116,028\times B‐79,907\times C+700\times AA\\ & +374\times BB‐702\times CC+1,561\times AB+1,298\times AC‐149\times BC \end{array}$	5

**TABLE 4:** Variables involved in the Central Composite Design (CCD) and response obtained

Solvent		Palm oil										Sunflower oil									
Process		CSE					UASE					CSE					UASE				
Source		Sum of squares	*df*	Mean square	*F*‐Value	*p*‐Value	Sum of squares	*df*	Mean square	*F*‐Value	*p*‐Value	Sum of squares	*df*	Mean square	*F*‐Value	*p*‐Value	Sum of squares	*df*	Mean square	*F*‐Value	*p*‐Value
A: Temperature		2.2712E+10	1	2.2712E+10	41.96	.000	2.9332E+11	1	2.9332E+11	287.65	.000	1.7253E+10	1	1.7253E+10	50.08	.000	2.6829E+11	1	2.6829E+11	9.38	.012
B: Time		2.4131E+09	1	2.4131E+09	4.46	.061	3.9258E+10	1	3.9258E+10	38.50	.000	8.6258E+08	1	8.6258E+08	2.50	.145	1.1432E+11	1	1.1432E+11	4.00	.073
C: Solvent to leaves ratio		6.8934E+08	1	6.8934E+08	1.27	.285	2.8874E+10	1	2.8874E+10	28.32	.000	1.4420E+09	1	1.4420E+09	4.19	.068	6.1989E+10	1	6.1989E+10	2.17	.172
AA		9.3966E+08	1	9.3966E+08	1.74	.217	9.2381E+10	1	9.2381E+10	90.59	.000	3.7077E+09	1	3.7077E+09	10.76	.008	7.0567E+10	1	7.0567E+10	2.47	.147
BB		17964E+09	1	1.7964E+09	3.32	.098	1.5059E+09	1	1.5059E+09	14.77	.003	8.9515E+06	1	8.9515E+06	0.03	.875	1.0195E+11	1	1.0195E+11	3.57	.088
CC		9.2879E+08	1	9.2879E+08	1.72	.220	1.1020E+08	1	1.1020E+08	10.81	.008	6.7752E+06	1	6.7752E+06	0.02	.891	1.8175E+09	1	1.8175E+09	0.06	.806
AB		7.4609E+07	1	7.4609E+07	0.14	.718	3.6282E+10	1	3.6282E+10	35.58	.000	8.2763E+06	1	8.2763E+06	0.02	.880	4.3881E+11	1	4.3881E+11	15.35	.003
AC		7.7356E+08	1	7.7356E+08	1.43	.259	6.4639E+05	1	6.4639E+05	0.00	.966	9.1172E+07	1	9.1172E+07	0.26	.618	2.1573E+10	1	2.1573E+10	0.75	.405
BC		5.3950E+07	1	5.3950E+07	0.10	.759	2.0040E+06	1	2.0040E+06	0.00	.980	3.0949E+07	1	3.0949E+07	0.09	.771	6.4075E+08	1	6.4075E+08	0.02	.884
Lack of fit		3.6290E+09	5	7.2579E+08	2.03	.227	7.5989E+09	5	1.5198E+09	2.92	.132	3.0364E+09	5	6.0728 E+08	7.43	.023	2.7327E+11	5	5.4654 E+10	21.67	.002
Pure error		1.7838E+09	5	3.5675 E+08			1.78385E+09	5	5.1969E+08			4.0893 E+08	5	8.1785 E+07			1.2608 E+10	5	2.5216 E+09		
Total (corr.)		3.5212E+10	19				5.1346E+11	19				2.6931E+10	19				1.3580E+12	19			
*R* ^2^	84.63		$R_{\text{adj}}^{2}$	70.79			98.01	$R_{\text{adj}}^{2}$	96.23			87.21	$R_{\text{adj}}^{2}$	75.69			84.63	$R_{\text{adj}}^{2}$	70.79		

### 3.2 Optimized conditions

The optimized parameters for UASE and CSE processes are tabulated in Table 5. This study found that the UASE process gives five times higher maximum citral area than the CSE process. The optimum temperature is at 81.8°C, and the optimum extraction time is 55.2 min except for lemongrass‐infused palm oil after the CSE process (45 min). The optimum solvent to leaves ratio is varied from 5.3:1 to 12.9:1.

**TABLE 5:** Summary of optimized parameter

Type of oil	Technique	Maximum citral area	Optimum temperature (^o^C)	Optimum extraction time (min)	Optimum solvent to leaves ratio
Palm oil	UASE	1.009 × 10^6^	81.8	55.2	5.3:1
	CSE	2.025 ×10^5^	81.8	45	5.4:1
Sunflower oil	UASE	1.767 × 10^6^	81.8	55.2	12.9:1
	CSE	2.179 × 10^5^	81.8	55.2	5.3:1

### 3.3 Characterization of the infused oils

**FIGURE 5:**
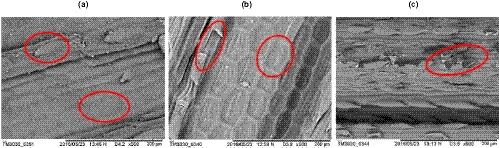
SEM micrographs of the lemongrass leaf: (a) virgin, (b) after CSE, and (c) after UASE (×500)

To study the oil content, three types of analysis were done which are Scanning Electron Microscopy (SEM), Fourier Transform Infrared (FT‐IR) Spectroscopy and Gas Chromatography‐Mass Spectrometry (GC‐MS). The lemongrass's morphological characteristics, before and after CSE and UASE processes, were illustrated in Figure 5. On the leaf's upper surface in Figure 5a, the body consists of prickle hair known as trichomes (Kaur & Dutt, 2013) and fixed shape Parenchyma cell, which is both in the circle. Due to CSE extraction's high temperature, the trichomes were ruptured, and the Parenchyma cells were shrunken compared to the fresh lemongrass leaf (Figure 5b). The trichomes that contain the metabolite liquid are the source of lemongrass oil. Whenever the trichomes break, the lemongrass oil is produced, and this figure demonstrates that the extraction process occurred. The lemongrass leaf's cell and trichomes that underwent the UASE process (Figure 5c) were ruptured and abraded, which might be due to the cavitation bubble induced by the ultrasonicator collapsing on the surface of the lemongrass leaf. This finding is consistent with the work as reported by Petigny et al. (2013) and Parniakov et al. (2015).

**FIGURE 6:**
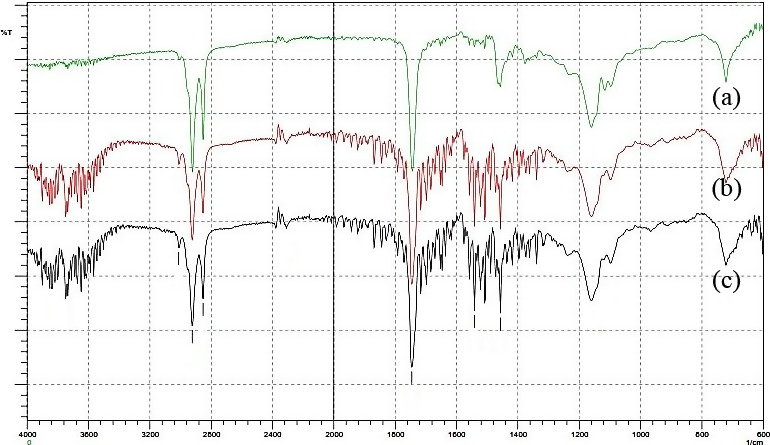
FT‐IR spectrum of three different types of oil samples; (a) palm oil, (b) sunflower oil, and (c) corn oil

Figure 6 shows the FT‐IR spectrum of extracted oil samples attained in the wavenumber region between 4,000 and 600 cm^−1^. The result from FT‐IR is tabulated in Table 6. It was found that the spectra of the three types of extracted oils were very much similar to each other. This indicates that the functional group of all cooking oil samples is not much different from each other. In vibrations at 3,010.88 cm^−1^, a stretching of =C‐H is observed corresponding to an alkene. Symmetric and asymmetric stretching of C‐H is observed in vibration at 2,922.16 and 2,852.72 cm^−1^. The intense band observed at 1,745.58 cm^−1^ is due to vibrations of C=O, which showed the presence of esters of saturated aliphatic. This group is also known as triglyceride (TGA), the dominant component in fats and oils (Wahab et al., 2015). The peak at 1,541.12 cm^−1^ indicates stretching of C=C of the alkenes group. The peak at 1,456.26 cm^−1^ is observed due to C‐C stretching, which is in the aromatics group. FT‐IR analysed each sample for all multivariate study. However, there is no significant change in the functional group after CSE and UASE were carried out for all cooking oil types. This is because all of the samples still contain the same functional group, organic functional groups. Other than that, it also shows that the component in the cooking oils did not change significantly.

**TABLE 6:** Results from FT‐IR analysis

	Palm oil peak (cm^−1^)	Sunflower oil peak (cm^−1^)	Corn oil peak (cm^−1^)
Stretching of =C‐H	3,005.10	3,010.88	3,010.88
Symmetric and Asymmetric stretching of C‐H	2,922.16 2,852.72	2,922.16 2,852.72	2,924.09 2,852.72
Triglyceride (TGA)	1,743.65	1,745.58	1,745.58
Stretching of C=C	1,510.66	1,541.12	1,541.12
Stretching of C‐C	1,460.11	1,456.26	1,456.26

Studies from GC‐MS revealed that both CSE and UASE processes produced infused oil that has similar components. Figure S1 shows the GC‐MS spectrum before the extraction process (virgin oil) and after the extraction process using palm oil, sunflower oil and corn oil. From the GC‐MS spectrum comparison between the virgin oil and infused oil, the additional component that had been ingrained from the lemongrass leaves are shown in Table 7. The graphs for corn oil on virgin and infused show no additional component appeared after the CSE and UASE processes. Hence, it can be concluded that corn oil cannot be used as a solvent to extract lemongrass oil. This may due to the component contained in the corn oil itself, which inhibit the extraction process.

**TABLE 7:** Lemongrass oil composition in infused palm oil and sunflower oil

Peak	Palm oil			Sunflower oil		
	Retention time	Compound	Relative peak area (%)	Retention time	Compound	Relative peak area (%)
a	8.649	Neral	0.97	8.628	Neral	7.67
b	9.075	Geranial	3.85	9.057	Geranial	19.44
c	18.541	9,12‐Octadecadienoic acid (Z,Z)‐	0.28	12.359	Decanoic acid, methyl ester	0.34
d	20.973	2H‐Pyran‐2‐one, tetrahydro‐6‐octyl	0.19	28.116	2,6,10,14‐Hexadecatetraenoic acid	8.08

## 4 CONCLUSIONS

This study found that the UASE process gives five times higher maximum citral area than the CSE process. The optimum temperature is at 81.8°C, and the optimum extraction time is 55.2 min except for lemongrass‐infused palm oil after the CSE process (45 min). The optimum solvent to leaves ratio is varied from 5.3:1 to 12.9:1. The SEM analysis shows that the lemongrass leaf consists of hair like that containing metabolism of the plant, which is also known as an essential oil. After going through the extraction process, the trichome was ruptured, thus produced the lemongrass oil. This study concludes that ultrasound‐assisted could assist the extraction process by induced cavitation bubbles and accelerates the release of organic compounds within the plant body. Thus, oil production is increasing, and the yield of oil production will be high. The FT‐IR Spectroscopy shows that the saturated aliphatic and aromatics group is present in the alkenes' infused oil. These entire groups give a significant peak in the FT‐IR spectrum. The GC‐MS analysis reveals the additional component in the infused oil. This study discovers that neral and geranial are the significant components added from the lemongrass.

## CONFLICT OF INTEREST

The authors declare that they have no conflicts of interests.

## Supporting information

Supplementary Material
