# Supplementary material for: Comparison and optimization of conventional and ultrasound‐assisted solvent extraction for synthetization of lemongrass (Cymbopogon)‐infused cooking oil
Source: Food Sci Nutr. 2021 Mar 22;9(5):2722–32. doi: 10.1002/fsn3.2234 (PMC8116846; doi:10.1002/fsn3.2234)
Supplement: Supplementary file 1 — Supplementary Material [file FSN3-9-2722-s001.docx]

**Supplementary Section**

**Table S1 Optimum condition plot of citral area under different experimental conditions**

| **Solvent** | **Process** | **Optimum Condition Plot** |
| --- | --- | --- |
| **Palm Oil** | **CSE** |  |
|  | **UASE** |  |
| **Sunflower Oil** | **CSE** |  |
|  | **UASE** |  |

|  | **Virgin Oil** | **Infused Oil** |
| --- | --- | --- |
| **Palm Oil** | **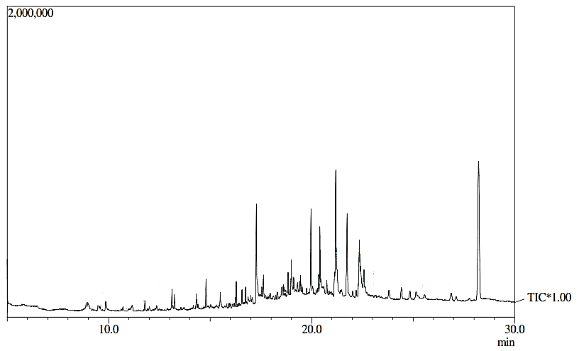** | 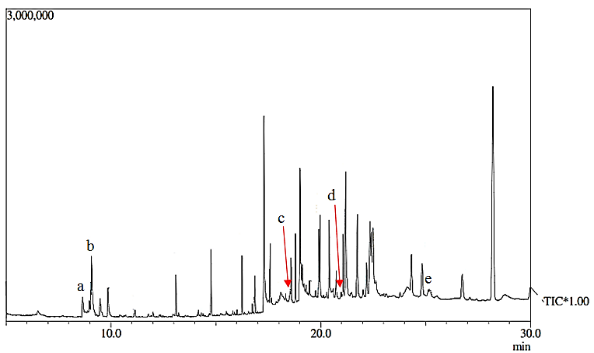 |
| **Sunflower Oil** | 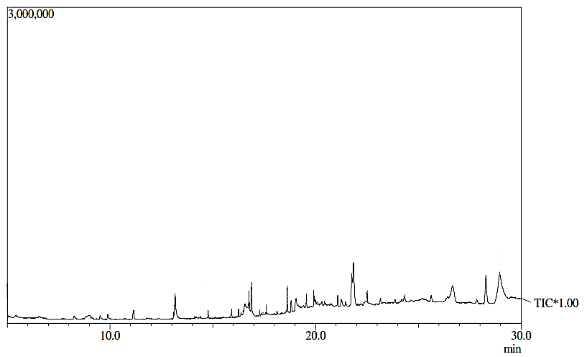 | 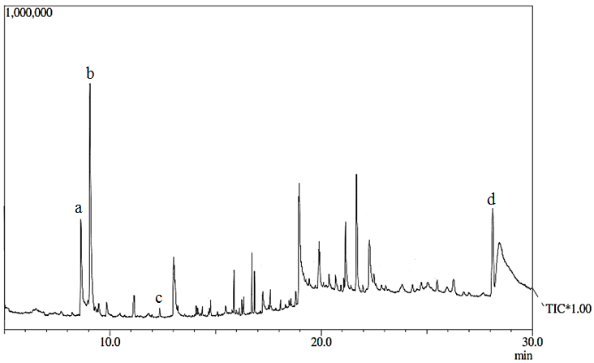 |
| **Corn Oil** | 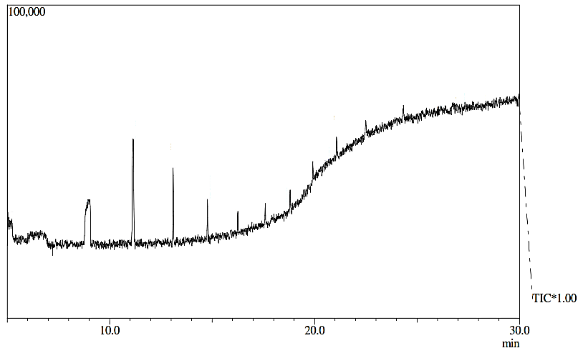 | 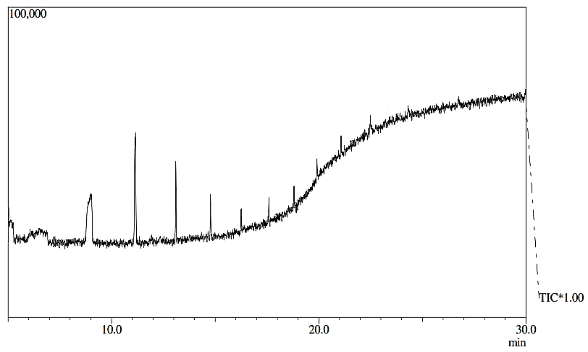 |

**Figure S1** GC-MS spectrum of palm oil, sunflower oil, and corn oil for both virgin and infused oils.
